# Investigating Facilitators and Barriers for Active Breaks among Secondary School Students: Formative Evaluation of Teachers and Students

**DOI:** 10.3390/children11020155

**Published:** 2024-01-25

**Authors:** Alice Masini, Giulia Longo, Matteo Ricci, Lawrence M. Scheier, Alessandra Sansavini, Andrea Ceciliani, Laura Dallolio

**Affiliations:** 1 Department of Translational Medicine, University of Eastern Piedmont, 28100 Novara, Italy; alice.masini@uniupo.it; 2 Department of Biomedical and Neuromotor Sciences, Section of Hygiene and Preventive Medicine, University of Bologna, 40126 Bologna, Italy; giulia.longo9@studio.unibo.it (G.L.); matteo.ricci18@studio.unibo.it (M.R.); laura.dallolio@unibo.it (L.D.); 3 LARS Research Institute, Inc., Sun City, AZ 85351, USA; 4 Prevention Strategies, Greensboro, NC 27410, USA; 5 Department of Psychology “Renzo Canestrari”, University of Bologna, 40127 Bologna, Italy; alessandra.sansavini@unibo.it; 6 Department of Life Quality Studies, University of Bologna, Campus of Rimini, 47921 Rimini, Italy; andrea.ceciliani@unibo.it

**Keywords:** physical activity, focus groups, adolescent, teachers, grounded theory, facilitators, barriers

## Abstract

Physical activity in the form of “active breaks” can be combined with academic instruction in primary school. However, few studies have examined the feasibility of conducting active breaks in secondary school. To address this gap, we conducted focus groups (FGs) regarding the implementation of an active breaks (ABs) protocol with 20 teachers and 10 secondary school students. Barriers/facilitators toward the implementation of ABs were classified using grounded theory inductive methods framed by the socio-ecological model. Individual-level factors were instrumental for both teachers and students. Teachers highlighted personal fears and concerns regarding using ABs, while students reported fears related to peer behaviour during the activity. Both teachers and students agreed that ABs can improve cognitive skills and time-on-task behaviour. Teachers articulated concerns related to student behaviour during ABs including possible social exclusion and injury. Students felt that ABs might affect classroom management and interfere with maintaining students’ academic focus. Teachers underscored that ABs required social support from the administration and colleagues. Students felt that ABs could support teachers’ instructional focus and provide them with an energy respite. Collectively, the FGs suggested that environmental limitations could hinder the implementation of ABs. Involving teacher and student feedback during the codesign phase can rationally inform the design of school-based ABs.

## 1. Introduction

A compilation of evidence accumulated from systematic reviews [1,2] and published guidelines [3] all reinforce the value of physical activity (PA) for children in terms of both current and future health. Among the myriad of benefits, research shows that PA promotes better sleep, psychological health (e.g., reduced depression), and well-being including reductions in some forms of anxiety [4,5]. At the scholastic level, PA improves academic performance and has been shown to improve cognitive functioning including performance on neuropsychological tests such as those involving mental processing speed and memory [5]. There are also social [6] and physical health benefits, the former including higher self-esteem and more social interactions (i.e., through sports participation) while the latter includes reduced occurrence of cardiovascular and metabolic disease [7]. Worldwide guidelines suggest that children and adolescents (from 5 to 18 years of age) should perform at least 60 min of moderate to vigorous physical activity (MVPA) daily in order to achieve the physical, mental, cognitive, and social benefits reported in the literature [4]. Despite the weight of evidence and the intrinsic value of published guidelines, the majority of children worldwide do not meet the recommended levels of PA and are characteristically labelled “physically inactive.” The observation of declining activity is troublesome because physical inactivity (PI) is a risk factor for premature mortality and several noncommunicable diseases [5], prompting some to consider PI as a new type of health pandemic. Addressing this concern through preventive efforts makes it imperative to increase PA among children and adolescents.

### 1.1. Sedentary Behaviour in Children

Physical inactivity may not be the only concern that affects the well-being of children and adolescents. This is because in addition to PI, children and adolescents are spending increasingly more time engaged in both screen-based (e.g., TV, computers, smartphones, videogames) or non-screen-based (e.g., reading a book, paper-based homework, playing board games) sedentary behaviours [8]. Sedentary behaviour is defined as any waking behaviour characterised by an energy expenditure of 1.5 METS or lower while sitting, reclining, or lying. In children, screen-based sedentary behaviours are associated with a range of poor health outcomes including overweight and obese conditions characterised by excessive body mass index (BMI) [9], cardio-metabolic risk, poor behavioural conduct, lack of fitness, and inadequate sleep [10]. Prolonged screen time, for instance, involving excessive use of a computer to play games, has been associated with unhealthy outcomes such as increased risk of type 2 diabetes, cardiovascular disease, all-cause mortality, and depressive symptoms [11,12]. Just as with PI and the lack of reinforcement for PA, the body of evidence supporting linkages between screen-based sedentary behaviours and poor health outcomes is noted worldwide [10]. As a result, many countries have started to include specific guidelines and recommendations for children and adolescents in order to address sedentary lifestyles. Collectively, these guidelines suggest breaking up periods of prolonged sedentary behaviour with periodic exercise and limiting recreational screen time [4]. A recently published set of guidelines [13] suggest that a healthy school day should include intermittent breaks to disrupt sedentary behaviour with breaks occurring every 30 min for children (5 to 11 years of age) and at least one time each hour for adolescents (12 to 18 years of age). The same guidelines suggest incorporating different types of movement as part of classroom activities and emphasizing their instructional and health value (e.g., teaching multiplication tables while engaging in physical movement; blending high-intensity interval training exercises and cognitive games, etc.). In this scenario, active breaks (ABs) consisting of 10–15 min of PA during school time represent an ideal strategy to counteract sedentary behaviour and improve PA levels [14,15,16]

### 1.2. Benefits of PA-Based Interventions

To a large degree, the body of evidence regarding integration of PA-based activity in the classroom and its effects on performance has been based on younger primary school children [14,15]. Logistically speaking, the structure of primary school lends itself to implementing health promotion programs. For instance, children remain with the same teacher throughout the school day, rather than changing instructors by subject matter. As a result, the same teacher covers a wide range of academic subjects and can intersperse PA with any subject. The same thing cannot be said about secondary school children, who may move from one class to another and have different teachers for different subjects. This makes it harder to schedule ABs with secondary school children. As a result, we know much less about the use of AB interventions with older secondary school youth (ages 11, 12 and 13), where the literature is either absent or not as conclusive [14]. Recently, Fenesi et al. suggested a wide range of challenges and misconceptions that could potentially affect conducting research on PA with secondary school youth [17]. The most prominent of these include structural (physical layout), classroom management (developmental factors), logistical (scheduling and curricular), and health literacy, all of which can interfere with a teacher’s ability and/or motivation to engage PA in a classroom setting. The fact that we know so little about the role of PA in adolescent development, a population that is at risk for sedentary and inactive behaviour, limits our ability to intervene with this age group. Conceivably, there are a myriad of ways that engaging in PA could benefit youth, perhaps in the same ways and achieving the same goals as with children. It is in this context that the present study aimed to examine the feasibility of including ABs in a secondary school setting. The goal of the study was to obtain information that could point to the required directions that would support the implementation of ABs in this age group. Prior to actually implementing ABs in a secondary school context, we conducted a series of focus groups with both teachers and students. We intended to use this information to identify factors that could impede or facilitate the implementation and sustainability of an ABs program when conducted in secondary schools.

### 1.3. Benefits of Formative Studies

Qualitative studies often provide more detailed insight and capture personal nuances that may be missed when relying solely on quantitative surveys. This is because self-report surveys with fixed responses often limit a person’s ability to expand, in a more descriptive fashion, on their feelings or sentiments [18]. One popular qualitative research tool involves conducting focus groups, which consist of small group discussions led by a moderator. During the group, participants respond to probes that elicit their thoughts, beliefs, and attitudes toward a particular topic [19]. This approach has been used extensively in market research to learn about how consumers feel about a particular product [19,20]. Extensions of this strategy include learning more about people’s opinions, attitudes, and beliefs toward health promotion [21] and their perceptions of prevention programs more generally [22]. Focus groups represent a “middle ground,” occupying a position between in-depth stakeholder interviews, which can provide detailed information from a single individual and participant observation [23], the latter avoiding direct interaction with the individuals [24]. The ability to mine the thoughts and feelings of several people during a free-flowing conversation enables a richer appreciation of how people think, process information, and interpret the world they occupy. In the context of health promotion settings, a goal of focus groups is to generate ideas about newly devised programs and also determine whether a program will “fit” the needs of a particular community or setting. This type of consumer information is a mainstay of many current health promotion programs and can involve utilizing codesign practices from the ground up [25,26]. It is with this in mind that we attempted to learn more about the “fit” of a physical activity program by drilling down deeper into what teachers and students perceive as barriers and facilitators to using ABs in regular classrooms. For the most part, and with few exceptions (e.g., music teachers who use movement integrated into their lessons), Italian teachers do not readily possess a great deal of knowledge about how to use ABs as part of their instruction. To address this gap, we conducted focus groups with both teachers and students to explore their beliefs and attitudes toward the practice of PA in daily school life. The study comes on the heels of recent evidence that shows this approach to be informative for PA-based interventions [27,28].

### 1.4. The Social Ecology Model

One of the required elements of using focus groups is a need to provide an “organizing framework” for the participant discussion [29]. This helps to tie the discussion to the main tenets of health promotion and provides a basis to probe different barriers and facilitators that can affect implementing or engaging in PA. Consider that an individual’s motivation to engage in PA can derive from several sources. On the one hand, they can channel family and parental influences [30,31]. For example, a youth whose parents are active and who encourage family activities may be more likely to engage in PA of his or her own volition. This type of “intergenerational transmission” of family values can promote the development of favourable beliefs and attitudes conducive to participating in sports and other PA. Likewise, communities can support PA through the addition of parks and bike lanes and conducting neighbourhood festivities (i.e., 5k runs) encouraging PA, among many other opportunities. Schools may also vary in the way they promote PA, with different social norms reflecting access to resources, the composition of the administration, and other organisational factors (i.e., history) that can act as facilitators or impediments to student participation in PA. Reliance on an overarching framework can help to create systematic and meaningful linkages between the development of an AB program and the feedback received from focus group participants. This type of structured framework can ensure that efforts to design and implement an AB intervention are developmentally and ecologically valid.

**Figure 1:**
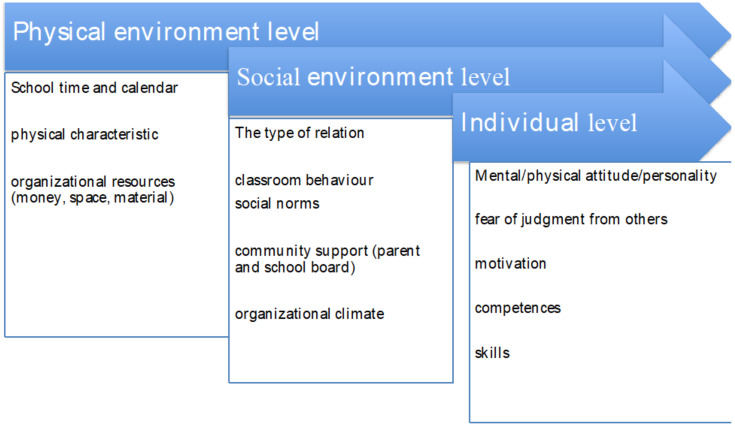
Three levels of SEM.

The social–ecological model (SEM) provides a heuristic framework for this purpose, primarily because it considers a multitude of “nested” levels of influence that can affect individual behaviour. Originally proposed by Bronfenbrenner [32] to account for the ecology of human development, the SEM posits that individuals reside at the interface of different organised “systems”, with each system exerting an influence on a person’s behaviour. According to SEM, the choice to pursue a healthy lifestyle revolves around a compilation of intrapersonal, interpersonal, institutional, community, and structural (policy) factors [33]. Each of these levels exerts some type of influence that contributes to shaping human behaviour. The different levels (representing “ecologies”) do not represent passive influences in the way they can promote or retard health but rather represent active forces that interact with other levels to empower individuals to act. Simply stated, an individual will not only engage in healthy behaviours on the sole basis of internal motivations (i.e., their personal valuation of health, knowledge, developmental history, or cognitions) but will also consider contextual influences that may or may not be conducive, social norms that operate in their community, and structural factors that can prevent healthy pursuits (i.e., access to resources, restrictive environments, culture, and economic factors). Collectively, all of these levels bear on a person’s decision making because individuals are embedded in and interact socially with other individuals at each level [34]. To be effective, an intervention needs to take into consideration that change, in the case of health promotion activities, comes about from understanding the nature of these social and structural relationships, their mutual exchange (i.e., spillover effects), and how change at one level can influence what transpires at another level. Increasingly, the SEM is being utilised to determine the fit of health promotion programs and acquire information on a program’s perceived utility and feasibility [35]. Figure 1 graphically portrays the three levels of SEM influence that guided the current focus group activities.

## 2. Materials and Methods

### 2.1. Study Design and Setting

The present work constitutes Phase 1 of a larger AB study (the BRAVE Study), which was intended to determine the effectiveness of an active breaks protocol implemented in a secondary school setting. Conductance of the focus groups comports with the Standards for Reporting Qualitative Research (SRQR) checklist. The study was conducted in two secondary schools located in a province of Bologna, Italy (Bazzano and Monteveglio). Other than being a teacher or student, there were no exclusion and inclusion criteria imposed for focus group participation. The study was approved by the two respective school boards, endorsed by the University of Bologna (Italy), approved by the University of Bologna Bioethics Committee on 18 March 2022 (Protocol n. 63053), and followed the Declaration of Helsinki. We enrolled teachers using invitation letters sent by the school principal to identify interested teachers with varying teaching experience in different subjects (i.e., math, science, literature, physical education, and music). Each teacher then selected two students from their classes in either the second or third grades (ages 12 to 13).

#### Participants

Focus groups consisted of no more than eight individuals each (students or teachers) and were conducted using open-ended prompts introduced by the moderator. The COVID-19 pandemic necessitated conducting the teacher focus groups online using the Microsoft Teams synchronous real-time communication platform. The students’ focus groups were conducted at school in a computer laboratory with all of the participating students present in the same room. Each student occupied a computer desk with headphones and a monitor to observe the moderator (who was virtual and not present in the school). Parents provided written informed consent for their children to participate in the focus groups. The only personal information collected from the student participants was their first name. For privacy reasons, the student focus group was not video recorded but only audio recorded and later transcribed. The focus groups with teachers were video recorded after obtaining written consent from all participants. At the beginning of each group, the moderator discussed ground rules, ensuring that participants were aware of the need for confidentiality, using first names only, that there were no right or wrong answers, and that participants should respect each other’s beliefs and opinions. For the student group, the moderator emphasized they should speak freely about their experiences and motivations and not only say what the moderator wanted to hear.

### 2.2. Procedures, Data Collection, and Analysis

The focus group probes were developed in collaboration with a psychologist (AS) and a pedagogist (AC). Unseen by any participants, two observers also attended the online focus groups in addition to the moderator. The moderator (AM) began the focus group by introducing a general “ice breaker” question, which helped the group obtain some familiarity with each other. The observers (LD and AC, not present in the room with the students or teachers) took notes on the participants’ expressions and their intonation (i.e., emphasis on words). In addition, two medical residents from the University of Bologna program in Hygiene and Public Health were present on the Team’s call and took notes. Because the groups were conducted online (asynchronous format), only the active speaker was visually available to each member (and the observers). Thematic saturation was achieved on a particular subject when participants could not generate any further information. We used qualitative content analysis, which is a flexible and dynamic strategy to analyse the focus group content. At each stage of the process, discussions among the research team helped identify key issues in the transcripts of the audio or video recordings. Three members of the investigative team copiously read each transcript line by line and coded the information inductively [36]. This involved collapsing the notes from medical residents, observers, and the moderator into a single cohesive document. At that point, and after carefully reading through the transcripts, the investigative team created descriptive themes grouping similar concepts (e.g., individual, physical, and social, and then within each category, barrier vs. facilitators). Two researchers coded the data independently, and the third researcher was involved in the discussion to reach a consensus. Final themes were generated after further discussions and deliberations between all of the team to ensure that there was thematic saturation [37]. All of the coding and derivation of subthemes were guided by the SEM [38]. Table 1A,B outlines the probes used in the teacher and student focus groups, respectively.

## 3. Results

A total of 20 teachers attended the two focus groups (18 females and 2 males from two secondary schools). Italian literature (n = 6) was the most prevalent subject taught among the teachers, followed by languages (Spanish and English, n = 4); math and science, physical education, and music were equally represented (n = 2), while art was the least represented (n = 1). Teachers ranged in years of employment experience from 1 to 36 years (12.95 ± 9.08). The student focus group consisted of six female and four male students (11–12 years of age). Key themes and subthemes that emerged from the data pertaining to barriers and facilitators toward the inclusion of AB intervention in secondary school children were classified according to the three SEM levels. Table 2 contains the final set of summarised themes for both teachers and students.

### 3.1. Results of Teacher Focus Groups

When addressing perceived barriers at the individual level, teachers mentioned that embarrassment was a factor (feeling ridiculous), as was feeling inadequate, and feeling too shy to demonstrate the activities. They also mentioned not having the proper knowledge to manage the intervention, lacking competence, and fear of not being able to control the class. Time wasting and being able to stick with their curricular mandates were also concerns. Interestingly, they also mentioned concerns with self-presentation, in other words, would students “see them” in a different light than their usual subject instructional mode? For facilitators, teachers mentioned personal motivation and letting them try the activities before using them with students. They also mentioned not holding any prejudices against PA and knowing there was administrative support for their efforts.

At the social level (e.g., interpersonal relations), when it came to barriers, teachers mentioned the potential for children to hurt themselves and the fact that students might feel inadequate or suffer psychologically if they cannot complete the exercises. They were also concerned that students would not see that exercise has any pedagogical value. Among the facilitators, teachers indicated that collaboration with the school board and with colleagues would help them prepare the activities, their participation in the ABs would strengthen the students’ motivation to participate, and that PA-based activities could translate to other areas of both academic and non-academic functioning. Moreover, teachers believe that ABs can improve cohesion among students.

When addressing barriers at the physical environmental level (e.g., organisational resources including financing, physical characteristics, school calendar), teachers mentioned a lack of space (physical features of the building), too many obstacles in the classroom (e.g., seats), and limited time to conduct activities (e.g., scheduling and performing Abs indoors). Among the facilitators, teachers mentioned keeping movements simple (not requiring too much space), using static exercises (moving single parts of the body), and having sufficient outdoor spaces to perform Abs.

### 3.2. Results of Student Focus Groups

The focus group discussion for students was also framed by the SEM. Responding to individual-level barriers, students reported the potential difficulty in calming down after Abs (difficulty in shifting moods for the next lesson) and the lack of fun during the activity (waste of time and lack of enthusiasm), especially for those who are not motivated to perform PA. When asked about facilitators, the students mention the use of game-like exercises that can allow everybody to perform the Abs, even going as far as to suggest creating new exercises. Moreover, students felt that ABs provide a means of reducing extraneous time off tasks (e.g., being tired and lacking concentration). At the social level, students indicated barriers related to time-scheduling by their teachers (e.g., lack of time, fear of losing time) and classroom management by their teachers (e.g., difficulty in completing the exercise given the surroundings and controlling the activity to avoid chaos). With regard to facilitators, students noted that ABs are of short duration, can be used to transition from one lesson to another, and provide a respite from learning during the last hour of school. Interestingly, students also mentioned that teachers would benefit from ABs because they have instructional value and can be used to reinforce educational materials (i.e., lessons might be easier to explain using ABs and teachers can re-energise themselves before further engaging in instruction). Finally, at the physical level, students concurred with their teachers, suggesting that the physical features of the building might hinder conducting simple exercises that may not be easy to perform in small spaces.

### 3.3. Codesign Results

In addition to conducting the focus groups, we also asked teachers and students to provide input with respect to key elements of implementing (and sustaining) an AB program. This is part of a codesign process, which we felt would make the intervention more feasible and attractive come time for actual implementation. When asked about the dosage of Abs, both teachers and students suggested a mean duration of 5–10 min, delivered at the beginning or at the conclusion of a lesson, in the morning, as an ice breaker prior to class instruction, or when dictated by the students’ classroom behaviour (i.e., agitated and unruly students might need a respite). Teachers desired to create ABs that could support academic content and include exercises focused on wellness, postural/ergonomic, relaxation/stretching, concentration, and self-knowledge through proprioception. In contrast, students wanted moderate to vigorous intensity exercises involving, for example, muscle activation exercises (e.g., squats, push-ups). When considering the setting for conducting ABs, both teachers and students agreed that ABs should be conducted inside classrooms; however, if possible, outdoor spaces (e.g., school corridor or playground) might be more appropriate. Finally, both teachers and students provided creative responses to how ABs should be implemented (i.e., the performance of ABs). Most of the proposals focused on the use of instruments and objects during the ABs (e.g., TikTok, music, multimedia contents, multimedia whiteboard, YouTube) and that ABs could be integrated into the content of the language arts for learning English.

## 4. Discussion

The present qualitative study aimed to explore barriers and facilitators to using ABs in a classroom setting for Italian secondary students. The impetus for this study was provided by the lack of concrete evidence that ABs can be used with students in this age group, despite increasing evidence that physical activity and sports practice have tremendous benefits for youth [6].

While sporting events, athletic activities, and physical education lessons are usually conducted in group settings outside of the classroom (e.g., playground and school gym), there is a paucity of evidence for this age group that ABs can be used as part of classroom educational activities, which is the focus of the AB intervention. Finding out more about perceived “barriers and facilitators” from both teachers and students thus represents the first step in determining whether this approach is feasible and will be adopted if implemented.

We guided the focus group discussions using the SEM, which provides a multi-tiered framework linking several levels of influence on human behaviour. Collectively, the focus groups suggested that physical limitations in the environment could limit the potential of ABs to be implemented in secondary schools. For instance, both students and teachers commented that the layout of the classroom could hinder implementation, as well as the need to be outside, using larger spaces for conducting some PAs. Individual-level factors also played a significant role in the deliberations of both teachers and students. Teachers commented that fears and concerns regarding using ABs in the classroom play a central role in whether to adopt an AB program. This trepidation more than likely reflects their personality, perhaps pointing toward their reluctance to try something new that involves certain physical challenges (i.e., balance or movement). Teachers would have to demonstrate the movement to the students and some may be reluctant to participate. Students also noted the pivotal role played by “individual differences”, pointing to their peers’ motivation and whether people would equally value the intrinsic fun of PA. However, like their teachers, students also noted that ABs could potentially improve cognitive skills (attentional focus) and have tremendous educational application.

With respect to social factors, teachers felt that exercise could result in injury to students or performance issues that could have psychological ramifications. Students, on the other hand, considered how ABs might affect the teachers, including classroom management and scheduling issues that might interfere with academics. On the favourable side, teachers saw the benefits of social support from the administration and their colleagues to institute ABs. Students felt that ABs could benefit teachers by supporting their instructional focus and giving them time to catch their breath and recover energy while teaching.

Feedback from both teachers and students as part of a codesign reinforced the notion that it is always important to consider the ease with which a program is implemented (e.g., complex programs are dissuasive). That is, the adoption of an AB program has to fit with current capabilities, not require too much change, and be motivational on its own. Both teachers and students suggested the same duration and frequency of the ABs, also in line with the recent literature on PA for older youth [39]; however, they suggested using different intensities with a younger age group. Teachers preferred to include exercises characterised by low intensity mixed with academic content, while students suggested the inclusion of more high-intensity and strength exercises, the latter of which would reduce sedentary behaviour (time spent sitting in a chair).

### Limitations

There are several limitations of this study worth nothing. First, the sample for both the teachers and students was relatively small. This brings up the issue of how many focus groups (or attendees) are needed prior to achieving thematic saturation or reaching the point where no new information is provided. There are no hard and fast rules with respect to this issue; however, recent methodological work suggests only a handful of groups are needed [40]. Compared to a host of other qualitative studies, including a recent systematic review of weight management studies [35], our sample was in line with both the number of attendees and the number of focus groups. Even still, it is worth noting that additional numbers of focus groups might not net any additional insight or produce more relevant information [18]. Notwithstanding, the relatively small size of the focus groups, in part, reflected conducting the study during the COVID-19 pandemic, which basically shut down the education system in Italy. Moreover, the mixture of teachers can have a tremendous influence on the content of their discussions. For instance, many music and art teachers already blend PA in their instructional format, even in Italy, where this approach is quite popular. They use expressive movement to capture “feelings” that are then translated into music or some art form. Teachers in the current study represented a fairly robust blend of academic disciplines including math, history, science, and other subjects (i.e., language arts), representing disciplines that do not traditionally rely on movement. We also used a computer-mediated setting for conducting the focus groups, as opposed to the traditional face-to-face setting. Again, COVID-19 made face-to-face impossible; however, we felt there would be no loss of information with students or teachers using computers to link into the groups. Conducting the focus group in a computer laboratory with each student occupying a desk with headphones enabled them to think reflectively about the subject at hand and then contribute to the discussion without feeling their emotions or facial expressions are being evaluated by their peers (i.e., self-presentation). Obviously, an intrinsic limit of this type of study is represented by the fact that some students as well as teachers may not feel comfortable reporting comments or opinions that are clearly in contrast with colleagues and classmates. This limit can partly be overcome by the skill of the moderator who has the task of maintaining a calm and open atmosphere throughout the focus group.

In addition to professional experiences, culture may play a role in whether teachers or students are receptive to ABs in the classroom. Studies of this nature therefore require cross-validation in other settings where cultural forces may influence individual-level behaviours. Each school is different from the others, so it is necessary to take into account the type of institution and the number of students and teachers in each institute, as well as the urban or mountain location of the school. Studies of this nature should certainly be repeated in different contexts to investigate any differences.

We used the SEM to ‘frame’ our study and guide the focus group discussions. However, SEM provides only a framework to examine intersecting structures (i.e., ecologies) in a person’s life. It does not represent a true theory that can make specific predictions about how one ecology influences another. Future studies may want to frame studies of ABs in light of a specific theory that can help drive the content of interventions.

## 5. Conclusions

The present study allowed us to identify barriers and facilitators related to a school-based PA intervention from the viewpoints of both students and teachers. We were able to capitalise on the qualitative phase (including codesign) and use this information to formulate the contents and implementation strategy for the BRAVE Study. This ensured that the AB protocol would be easy to implement by teachers and at the same time would motivate students to participate and engage in PA during their classroom time.

## Figures and Tables

**Table 1:** (**A**) Key questions used as probes in the focus group discussions with teachers. (**B**) Key questions used as probes in the focus group discussions with students.

(A)	
Phase	Probes
**Opening probe to start the discussion** Exploring beliefs regarding the practice of physical activity as part of classroom activities Introducing the concept of “active breaks (ABs)”	Does physical activity play a role in your daily school life? Have you had any experience including physical activity as part of curricular lessons? What has this experience been like? Was it positive for you?
**Individual level** Exploring individual barriers and facilitators related to AB practice	Are there any personal barriers that might happen if you start practicing ABs in class? Can you provide some examples of personal issues that may prevent you from implementing ABs in the classroom? Do you feel you can adequately address these barriers? Are there things that you can do to facilitate the inclusion of ABs experiences in your classroom?
**Social environment level (teachers, children, principals, parents)** Exploring social environment barriers and facilitators related to AB practice	Are there social barriers involving certain people that might prevent use of ABs in the schools? Can you address these if you start practicing ABs in class? Can students facilitate the use of ABs in school? Do you think there are situations involving principals at the school that may hinder implementation of ABs? Do you think there are situations brought about by parents that could hinder use of ABs in school? Do you think there are situations that involve relations with particular people that make it possible to conduct ABs in school?
**Physical environmental level** Exploring physical and environmental barriers and facilitators to AB practice	Are there special features of the school, for example in the classroom, the playground or the gym that make it easy for you to use ABs in the school? What type of things in the physical environment might inhibit your ability to implement ABs? Do you feel that you can address any of these barriers, and if so, which ones?
**(B)**	
**Phase**	**Questions**
**Opening probe to start the discussion** Exploring beliefs regarding the inclusion of physical activity as part of classroom activities Introducing the concept of “active breaks (ABs)”	Do you engage in any physical activity during the regular school day? Have you had the opportunity to engage in physical activity while working in your classroom? What was this experience like? Did you enjoy it? If not, why? Would you look forward to including physical activity as part of your classroom activity (lessons)? If not, can you explain why?
**Individual level** Exploring person-level factors that can prevent using physical activity in the classroom	Are the factors that you think could make it difficult to include using physical activity in the classroom? What are some of these factors? Do you feel that you could benefit from including physical activity as part of classroom activities? Can you list any of these benefits? Do you think there are some disadvantages to including physical activity in the classroom? If so, what are some of the disadvantages?
**Social environment level (teachers, parents)** Exploring possible things that could prevent the use of physical activity in the classroom and that involve other people	Do you think that teachers can make it easier to use physical activity as part of classroom learning? Is it possible for parents to help out in using physical activity in the classroom? Are there things that may get in the way of teachers using physical activity in the classroom? What are some of these things? Are there things that parents might do to prevent your school (and teachers) from using physical activity as part of the classroom learning experience? Can you think of anything special that might get in the way?
**Physical environmental level** Exploring physical and environmental barriers and facilitators to AB practice	Which type of physical environment barriers might be encountered in your opinion? Are there things at your school that may make it easy to engage in physical activity in the classroom? For example, is your classroom big enough? Could you go outside to a playground? Can you use the gym? Are there any “props” that you can use that may make it fun to engage in physical activity in the classroom?

**Table 2:** (**a**) Socio-ecological analysis of barriers and facilitators—teachers. (**b**) Socio-ecological analysis of barriers and facilitators—students.

(a)	
Barriers	
**Individual**	Feeling ridiculous Losing authority Understanding what the task is and how to perform it Difficulties in presenting oneself (teacher) in a new and different guise Inadequacy Teacher’s personality/temper Lack of competence in PA Shyness Considering the activity as leisure time, in case the activity lacks a final goal
**Social**	Teasing (both towards the teacher and among students) Students may be disappointed if they fail the exercise (delusion) Respect for relationships between students Students could become hurt Social exclusion Not feeling comfortable with physical activity, psychological barriers Waste of time
**Environmental**	At which point in the lesson to insert the physical activity Limited time to organise activities Having very numerous classes It is difficult to stay on schedule Little time available to carry out the task Students are clumsy in their movements Lack of time to clearly explain the activity Having to wear masks Travel time (for outdoor activities) Tiny spaces Obstacles in the classroom (seats, desks) Difficulties in moving Risks related to the structure of the buildings (windows, etc.)
**Facilitators**	
**Individual**	Personal motivation No prejudices or mental barriers Experimenting with the activities before the beginning of the project (to convince teachers about its feasibility) Respect for personal timing, not forcing anybody
**Social**	Collaboration with the class council Involving all the teachers (together as a team) in designing the activity (it would be a positive message for the students) Having somebody as a support (at least at the beginning) to overcome psychological barriers Stating a common goal for teachers and students Setting up games during the lessons, using subjects and topics as tools Cohesion between teacher and pupils Overcoming limits as a game, together with the teacher
**Environmental**	Availability of outdoor spaces Empty/cleared environments Simple movements (not requiring a lot of space to perform) Trying “static” PA or moving only certain parts of the body (in this way, a small space is also manageable)
**(b)**	
**Facilitators**	
**Individual**	Taking a break during very long lessons Activity in pairs Letting off steam through the movement so as to be quiet afterwards Helpful against tiredness
**Social**	Teachers could have a rest during the active break While implementing ABs, teachers could relax and clear their heads It might be easier for the teacher to explain the lesson after the AB Short-duration ABs can be used to transition from one lesson to another
**Environmental**	Simple exercises easy to perform in small places
**Barriers**	
**Individual**	Difficulty in calming down Adjust mood for the next lesson Lack of fun
**Social**	Chaos Confusion Anxiety Opposition from those who do not enjoy ABs Difficulty in managing the ABs Teachers fear wasting part of their time dedicated to teaching
**Environmental**	Space difficulties

## Data Availability

All data and materials are available by written request to the corresponding author due to the population of children and adolescent involved in the focus group recordings.

## References

[1] Janssen I., Leblanc A.G. (2010). Systematic review of the health benefits of physical activity and fitness in school-aged children and youth. Int. J. Behav. Nutr. Phys. Act..

[2] Poitras V.J., Gray C.E., Borghese M.M., Carson V., Chaput J.P., Janssen I., Katzmarzyk P.T., Pate R.R., Connor Gorber S., Kho M.E. (2016). Systematic review of the relationships between objectively measured physical activity and health indicators in school-aged children and youth. Appl. Physiol. Nutr. Metab..

[3] Oja P., Bull F.C., Fogelholm M., Martin B.W. (2010). Physical activity recommendations for health: What should Europe do?. BMC Public Health.

[4] Bull F.C., Al-Ansari S.S., Biddle S., Borodulin K., Buman M.P., Cardon G., Carty C., Chaput J.P., Chastin S., Chou R. (2020). World Health Organization 2020 guidelines on physical activity and sedentary behaviour. Br. J. Sports Med..

[5] U.S. Department of Health and Human Services (2018). Physical Activity Guidelines for Americans.

[6] Eime R.M., Young J.A., Harvey J.T., Charity M.J., Payne W.R. (2013). A systematic review of the psychological and social benefits of participation in sport for children and adolescents: Informing development of a conceptual model of health through sport. Int. J. Behav. Nutr. Phys. Act..

[7] Teixeira P.J., Carraça E.V., Markland D., Silva M.N., Ryan R.M. (2012). Exercise, physical activity, and self-determination theory: A systematic review. Int. J. Behav. Nutr. Phys Act..

[8] Tremblay M.S., LeBlanc A.G., Kho M.E., Saunders T.J., Larouche R., Colley R.C., Goldfield G., Gorber S.C. (2011). Systematic review of sedentary behaviour and health indicators in school-aged children and youth. Int. J. Behav. Nutr. Phys Act..

[9] Cole T.J., Lobstein T. (2012). Extended international (IOTF) body mass index cut-offs for thinness, overweight and obesity. Pediatr. Obes..

[10] Carson V., Hunter S., Kuzik N., Gray C.E., Poitras V.J., Chaput J.P., Saunders T.J., Katzmarzyk P.T., Okely A.D., Connor Gorber S. (2016). Systematic review of sedentary behaviour and health indicators in school-aged children and youth: An update. Appl. Physiol. Nutr. Metab..

[11] Grøntved A., Hu F.B. (2011). Television viewing and risk of type 2 diabetes, cardiovascular disease, and all-cause mortality: A meta-analysis. JAMA.

[12] Grøntved A., Singhammer J., Froberg K., Møller N.C., Pan A., Pfeiffer K.A., Kristensen P.L. (2015). A prospective study of screen time in adolescence and depression symptoms in young adulthood. Prev. Med..

[13] Saunders T.J., Rollo S., Kuzik N., Demchenko I., Bélanger S., Brisson-Boivin K., Carson V., da Costa B.G.G., Davis M., Hornby S. (2022). International school-related sedentary behaviour recommendations for children and youth. Int. J. Behav. Nutr. Phys. Act..

[14] Masini A., Marini S., Gori D., Leoni E., Rochira A., Dallolio L. (2020). Evaluation of school-based interventions of active breaks in primary schools: A systematic review and meta-analysis. J. Sci. Med. Sport.

[15] Norris E., van Steen T., Direito A., Stamatakis E. (2020). Physically active lessons in schools and their impact on physical activity, educational, health and cognition outcomes: A systematic review and meta-analysis. Br. J. Sports Med..

[16] Sortwell A., O’Brien K., Murphy A., Ramirez-Campillo R., Piggott B., Hine G., Newton M. (2024). Effects of plyometric-based structured game active breaks on fundamental movement skills, muscular fitness, self-perception, and actual behaviour in primary school students. Biol. Sport.

[17] Fenesi B., Graham J.D., Crichton M., Ogrodnik M., Skinner J. (2022). Physical Activity in High School Classrooms: A Promising Avenue for Future Research. Int. J. Environ. Res. Public Health.

[18] Darbyshire P., Macdougall C., Schiller W. (2005). Multiple methods in qualitative research with children: More insight or just more?. Qual. Res..

[19] Kruger R.A., Casey M.A. (2015). Focus Groups: A Practical Guide for Applied Research.

[20] Stewart D.W., Shamdasani D.M. (2015). Focus Groups: Theory and Practice.

[21] Coverdale G.E., Long A.F. (2015). Emotional wellbeing and mental health: An exploration into health promotion in young people and families. Perspect Public Health.

[22] Scheier L.M., Kumpfer K.L., Brown J.L., Hu Q. (2019). Formative Evaluation to Build an Online Parenting Skills and Youth Drug Prevention Program: Mixed Methods Study. JMIR Form. Res..

[23] Morgan D.L. (1997). Focus Groups as Qualitative Research.

[24] Whyte W.F., Greenwood D.J., Lazes P. (1989). Participatory action research: Through practice to science in social research. ABS.

[25] Metzler C.W., Sanders M.R., Rusby J.C., Crowley R.N. (2012). Using consumer preference information to increase the reach and impact of media-based parenting interventions in a public health approach to parenting support. Behav. Ther..

[26] Moore S.K., Guarino H., Acosta M.C., Aronson I.D., Marsch L.A., Rosenblum A., Grabinski M.J., Turk D.C. (2013). Patients as collaborators: Using focus groups and feedback sessions to develop an interactive, web-based self-management intervention for chronic pain. Pain Med..

[27] Tay G.W.N., Chan M.J., Kembhavi G., Lim J., Rebello S.A., Ng H., Lin C., Shek L.P., Lança C., Müller-Riemenschneider F. (2021). Children’s perceptions of factors influencing their physical activity: A focus group study on primary school children. Int. J. Qual. Stud. Health Well-Being.

[28] Martínez-Andrés M., Bartolomé-Gutiérrez R., Rodríguez-Martín B., Pardo-Guijarro M.J., Garrido-Miguel M., Martínez-Vizcaíno V. (2020). Barriers and Facilitators to Leisure Physical Activity in Children: A Qualitative Approach Using the Socio-Ecological Model. Int. J. Environ. Res. Public Health.

[29] Sterdt E., Liersch S., Walter U. (2014). Correlates of physical activity of children and adolescents: A systematic review of reviews. HEJ.

[30] Lindsay A.C., Wasserman M., Muñoz M.A., Wallington S.F., Greaney M.L. (2018). Examining Influences of Parenting Styles and Practices on Physical Activity and Sedentary Behaviors in Latino Children in the United States: Integrative Review. JMIR Public Health Surveill..

[31] Moral-García J.E., Urchaga-Litago J.D., Ramos-Morcillo A.J., Maneiro R. (2020). Relationship of Parental Support on Healthy Habits, School Motivations and Academic Performance in Adolescents. Int. J. Environ. Res. Public Health.

[32] Bronfenbrenner U., Friedman S.L., Wachs T.D. (1999). Environments in developmental perspective: Theoretical and operational models. Measuring Environment across the Life Span: Emerging Methods and Concepts.

[33] McLeroy K.R., Bibeau D., Steckler A., Glanz K. (1988). An ecological perspective on health promotion programs. Health Educ. Q..

[34] Stokols D. (1996). Translating social ecological theory into guidelines for community health promotion. Am. J. Health Promot..

[35] Lang S., Gibson S., Ng K.W., Truby H. (2021). Understanding children and young people’s experiences pursuing weight loss maintenance using the Socio-ecological Model: A qualitative systematic literature review. Obes. Rev..

[36] Glaser B.G., Strauss A.L. (1967). The Discovery of Grounded Theory: Strategies for Qualitative Research.

[37] Strauss A., Corbin J. (1998). Basics of Qualitative Research.

[38] Golden S.D., Earp J.A. (2012). Social ecological approaches to individuals and their contexts: Twenty years of health education & behavior health promotion interventions. Health Educ. Behav..

[39] Masini A., Ceciliani A., Dallolio L., Gori D., Marini S. (2022). Evaluation of feasibility, effectiveness, and sustainability of school-based physical activity “active break” interventions in pre-adolescent and adolescent students: A systematic review. Can. J. Public Health.

[40] Guest G., Namey E., McKenna K. (2017). How many focus groups are enough? Building an evidence base for nonprobability sample sizes. Field Methods.

